# Complete mitogenome of the ixodid tick *Dermacentor reticulatus* (Acari: Ixodida)

**DOI:** 10.1080/23802359.2020.1821811

**Published:** 2020-09-16

**Authors:** Mikhail Yu Kartashov, Alexander N. Shvalov, Natalya L. Tupota, Vladimir N. Romanenko, Nina S. Moskvitina, Vladimir A. Ternovoi, Valery B. Loktev

**Affiliations:** a State Research Center of Virology and Biotechnology “Vector,” Rospotrebnadzor, World-Class Genomic Research Center for Biological Safety and Technological Independence, Koltsovo, Novosibirsk Region, Russia; b National Research Tomsk State University, Tomsk, Russia

**Keywords:** Tick, mitogenome, *Dermacentor reticulatus*, tick-borne infections

## Abstract

Here, we present the complete mitochondrial DNA sequence of *Dermacentor reticulatus*. The mitogenome is 14,806 bp and contains 13 protein-coding, 2 rRNA, and 22 tRNA genes, along with 2 control regions. *Dermacentor reticulatus* mitogenome has the common mitochondrial gene order of *Metastriata* ticks. It is phylogenetically close to the mitogenomes of *Dermacentor* ticks, of which *D. everestanus* mitogenome is the closest with 85.7% similarity. These data provide insights into the phylogenetic relations among *Dermacentor* ticks.

The tick *Dermacentor reticulatus* (Fabricius, 1794) is widely distributed in Europe and Northern Asia and is the principal transmission vector for tick-borne infections, such as those caused by tick-borne encephalitis virus as well as Omsk hemorrhagic fever viruses, *Rickettsia raoultii* and *Rickettsia slovaca* (Wójcik-Fatla et al. 2011; Biernat et al. 2014; Karan et al. 2014; Shchuchinova et al. 2015; Zając et al. 2017; Chitimia-Dobler et al. 2019). Additionally, the genetic markers of Kemerovo and bluetongue viruses, *Coxiella burneii, Bartonella quintana,* and *Theileria equi,* and different species of *Borrelia* spp., *Anaplasma s*pp, and *Babesia* spp have been detected in *D. reticulatus*. Recently, *D. reticulatus* has spread considerably and reached Siberian towns (Kartashov et al. 2019).

Here, we report the first complete mitochondrial DNA sequence of *D. reticulatus*. We collected adult ticks from a large town park in Tomsk (56°27’00.6“N 84°57'24.2“E) in 2017. Each tick was morphologically characterized and genetically identified with the sequencing fragment *cox1* as previously described (Kartashov et al. 2019). The ticks were individually frozen in liquid nitrogen, crushed using plastic pestles, homogenized in phosphate-buffered saline, and treated with TRIzol reagent (Invitrogen Co., USA) to extract their DNA. Sequencing was performed using a MiSeq Reagent Kit v3 for 400 cycles. Raw reads and *de novo* contigs were aligned to *D. everestianus* mitogenome without inversions or transitions by using BWA (v.0.7.15). Of the 2409 aligned paired-reads, the mean length was 143 bp, and the coverage for the resulting mitogenome was 46,8. Gene annotation and sequence analysis were carried out through BLAST searches that queried the complete mitochondrial genome sequence of *D. everestianus*. ARWEN (v1.2) software was used to predict transfer RNA (tRNA) genes and their secondary structures and was adjusted to other frames (Jühling et al. 2012). For phylogenetic analysis, the mitochondrial sequences were derived from NCBI and aligned using MAFFT with the default settings (Katoh and Standley 2013).

The complete mitogenome of *D. reticulatus* was estimated at 14,806 bp, encoding 13 protein, 2 rRNA, and 22 tRNA genes and found to contain 2 control regions (MT478096). The mitogenome encoded 3609 amino acids in total. The A + T contents of the protein-coding genes ranged from 71.54% (*cox1*) to 80.49% (*atp8*). The order of the genes in *D. reticulatus* mitogenome and their transcriptional directions were found identical to those of *D. marginatus*, *D. silvarum*, and *D. everestianus*. The overall base composition of *D. reticulatus* mitogenome was determined to be 39.8% T, 12.5% C, 38.6% A, and 9.2% G. A + T ratio ranged from 74.0% (control region 1) to 89.4% (tRNA-Gly). *D. reticulatus* mitogenome was found 84.8%, 85.1%, 85.4%, and 85.7% identical to those of *D. silvarum*, *D. marginatus*, *D. nutalii*, and *D. everestanus*, respectively. The translation of 6 protein-coding genes (*cox1*, *atp8*, *nd2*, *nd3*, *nd5*, and *nad6*) was predicted to be initiated by the ATT start codon, of 1 gene (*nad1*) by the ATA codon, and 6 genes by the classical ATG codon. It was observed that most of the protein-coding genes terminated with TAA, whereas the translation of 4 genes was predicted to be stopped by the incomplete stop codon T. The 12S and 16S rRNA genes were estimated at 697 and 1204 bp, respectively.

Additionally, we observed that the 22 tRNA genes ranged between 56 bp (tRNA-Ser) and 69 (tRNA-Met) bp. Based on their sequences, we predicted these tRNAs to have the typical cloverleaf secondary structure, except for tRNA-Cys and tRNA-Ser1 (anticodon TCT), which lack the D-arms. Control regions 1 and 2 were found to be 311 and 305 bp, located between 12S rRNA and tRNA*-*Ile genes, and between tRNA-Leu and tRNA-Cys, respectively. We identified 94 noncoding nucleotides in 12 unassigned intergenic regions and short overlaps that totaled 46 bp at 5 gene junctions, with the largest overlap (26 bp) at the junction of tRNA-Glu and *nd1*. Phylogenetic analysis revealed that *D. reticulatus* and *Dermacentor* species clustered together with high statistical support, indicating that *D. reticulatus* belonged to the genus *Dermacentor* (Figure 1).

**Figure 1.**
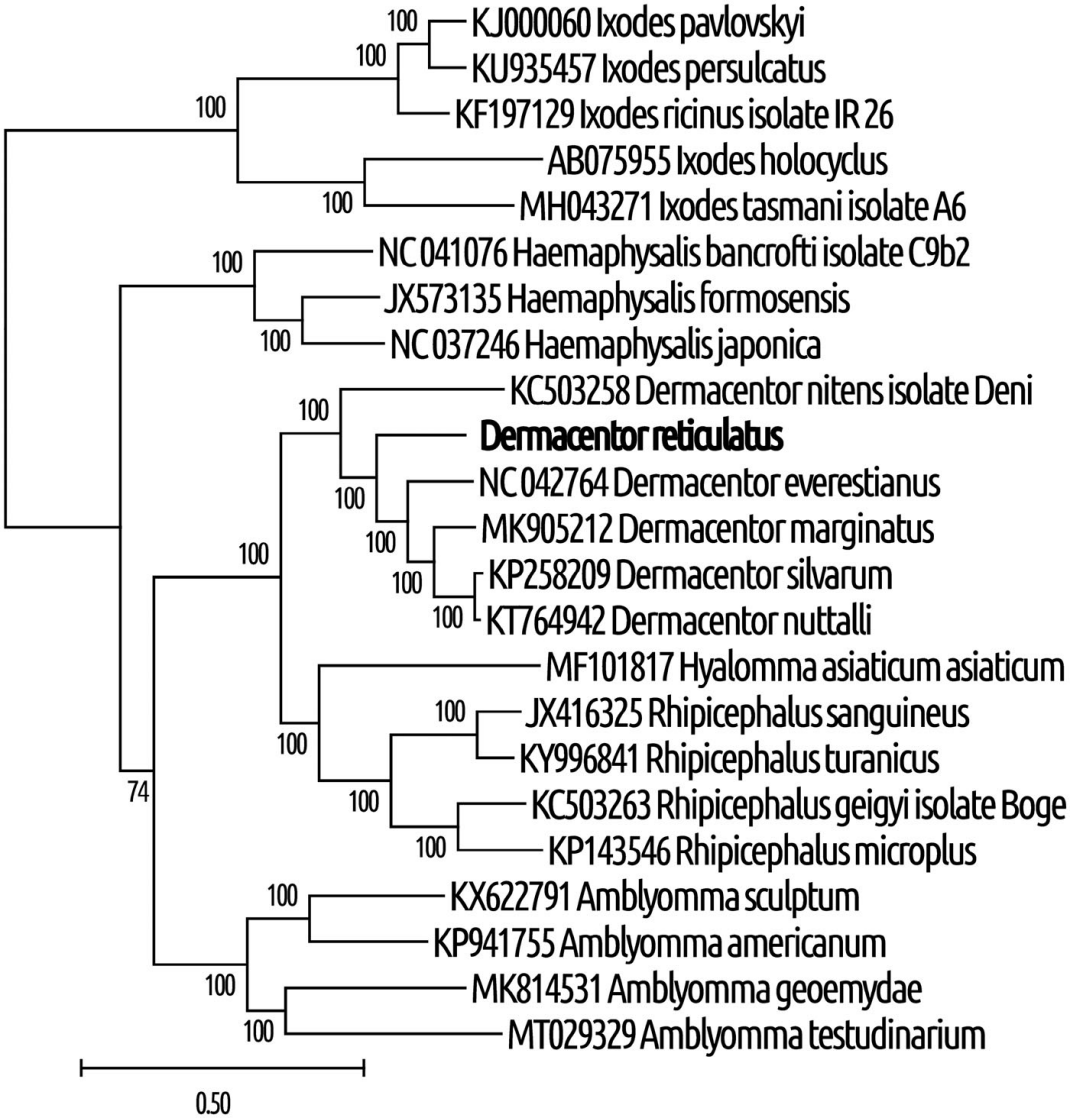
Phylogenetic relationships between *D. reticulatus* and other tick species, based on mitochondrial sequences. The method of maximal likelihood with the GTR (G + I) algorithm was used (Guindon et al. 2010). Support indices were defined using the bootstrap method with 1000 repeats. MEGA X software was used for visualization (Kumar et al. 2018). The GenBank accession numbers and species of ticks are indicated on the tree. The genetic distance scale is shown at the bottom. The bold type was sequenced in this study.

In conclusion, this study provides a new mitochondrion resource for phylogenetic studies and also novel genetic markers for further studies on *Dermacentor* ticks.

## Data Availability

The collected ticks *Dermacentor reticulatus* (Fabricius, 1794) was archived in Collections of Tomsk State University (Tomsk, Russia) registered on “The Insect and Spider Collections of the World Website” under collection number UTR II 595.421.1 (http://hbs.bishopmuseum.org/codens/codens-inst.html). The sequence data that support the findings of this study are openly available on GenBank using the accession number MT478096 (https://www.ncbi.nlm.nih.gov/nuccore/MT478096.1).
